# Graves’s Disease with and without Exophthalmic Goitre

**Published:** 1904-10

**Authors:** 


Graves's Disease With and Without Exophthalmic Goitre. By William Hanna Thomson, M.D., LL.D., Physician to the Roosevelt Hospital, New York. Octavo, pp. 143. New York: William Wood & Co. 1904. (Price, $1.50.)

The author's object is to emphasise the fact that the constitutional and general derangements which are characteristic of

Graves's disease constitute the disease, and not the condition of the thyroid gland or its accessories. The author's opinion is supported by a series of personal cases, with thyroid involvement on one hand and cases without thyroid involvement on the other, and comparisons made to show that the disease may have no recognisable and therefore probably no necessary relation to any state of the thyroid gland. The symptomatology and treatment of Graves's disease is gone into quite thoroughly and the use of a mercerised laxative strongly urged twice weekly. Tachycardia is regarded as the specific symptom, and exophthalmos and goitre as secondary manifestation.

The author's manner of proving his theory is very methodical and perhaps convincing; yet there are many who still agree with Möbius that the disease is secondary to thyroid morbid activity.

